# Report on Otology

**Published:** 1870-10

**Authors:** Samuel J. Jones

**Affiliations:** Chairman


ARTICLE XXXI.

### REPORT ON OTOLOGY.

BY SAMUEL J. JONES, A.M., M.D., Chairman.

The advance made in the last quarter of a century in the different branches of the profession of medicine, has given increased interest to those engaged in its pursuit.

To achieve this result, Science in general, has done much. New and improved instruments for diagnosis assisted in making visible, conditions which reasoning could only render probable, before the discovery of such apparatus.

The Stethoscope enabled the physician to explore a region, though hidden from view, with results almost as accurate as vision could have revealed. The Ophthalmoscope cleared away the mist that veiled diseases of the interior of the eye. The Laryngoscope, the Rhinoscope, and the Otoscope, have achieved results eminently satisfactory. The Endoscope, the Sphygmagraph, and others have aided in physical research. Although undue enthusiasm, may have claimed for these, a degree of importance that experience scarcely justifies, yet they have their use, and it should be the aim of the profession, instead of condemning them for their insufficiency, to endeavor by honest effort, to widen the sphere of their usefulness, and to so improve them as to increase the amount of good they are capable of achieving.

The advantages which resulted from the discovery of the Ophthalmoscope are recognized by all. What before, had been mysterious, began to become clear. A new interest attached to the study of diseases of the eye. The number of workers in, and of works on Ophthalmology rapidly multiplied, and many a grateful sufferer to-day, returns reverent thanks for such result.

One other department of our profession, though of recognized importance, remained devoid of interest—neglected—and the afflicted continued to suffer. Diseases of the ear continued to be prominent as among the opprobria of our profession. They were shunned by its members as difficult and uninteresting in diagnosis. The results of treatment were unsatisfactory in a marked degree. The unfortunate sufferer from these affections must either be left to nature to repair the injury, as best she might, or he must fall a prey to the empiric. An occasional spasmodic effort was made, at intervals running through years, to do something to confine aural practice to the medical profession, but the results were scarcely such as had been hoped for. It would not be interesting—scarcely profitable—to dwell upon the feeble efforts made in this direction, before the time of Sir William Wilde, of Dublin, whose work resulted in more accurate diagnosis, and with whom we may fairly estimate the beginning of aural surgery. With this as the initiative, Mr. Toynbee, of London, engaged in the same pursuit, with such energy and such a degree of success, as made a new era in that department of Surgery. His investigations, and the results of them, went far towards establishing accurate diagnosis; narrowed the field of doubt and uncertainty that had enveloped those diseases, and excited an interest in them that is every day becoming more extended. His success made him conspicuous, and amidst his career of usefulness, he fell a victim of accident. His work so nobly begun, has been pursued by Hinton, Yearsley, Anderson, and others of his countrymen, who were enabled to profit by his work, and to add to the result. Great Britain did not remain the only country that sought to widen this field of study. Germany, with that patient research which characterizes all her labors, betook herself anew to the work, and humanity may congratulate itself on the results attained by such men as Politzer, of Vienna, Gruber, of Vienna, Voltolini, of Breslau, Swartze, of Halle, Trötsch, of Würzburg, and Lucae, of Berlin — men whose lives are devoted to the investigation of these diseases, and their remedy.

Operative surgery of the eye seems to offer so much more attraction for the surgeon, that more who prefer special practice elect that; or, where diseases of the Eye and Ear are treated by the same person, diseases of the Ear, perhaps, receive a minor share of attention. This may be said of France and America, where Ophthalmology and Otology are more united than in either Great Britain or Germany.

In Paris, Löewenberg, in Boston, Clarke, in New York, Agnew, Roosa, Noyes, and Hackley, and in Chicago, Holmes and Hildreth have perhaps done as much as any others in their respective countries, to give aural surgery its proper degree of attention, and to show how much good may be done by faithful work in an unattractive and neglected, but important field of labor.

Having given a cursory view of workers in Otology, mention of a few of their most important works would seem to be proper; such as Sir William Wilde's "Aural Surgery;" "Diseases of the Ear," by Toynbee, with later edition of it by Hinton; "Trötsch on the Ear," translated by Roosa, and Politzer on the "Membrana Tympani," translated by Mathewson and Newton. Besides these works, there are published in Germany, the "Archiv für Ohrenheilkunde," a quarterly journal, and "Monadschrift für Ohrenheilkunde," a monthly, both devoted exclusively to diseases of the Ear; and in New York and Carlsruhe, simultaneously, the Archives of Ophthalmology and Otology, a quarterly, which constitutes the bulk of the authentic literature of that department at the present day.

In this connection it may be stated that two Otological Societies, one in the United States. and the other in Germany, have been organized, to foster the science of Otology, which [illegible]d annual meetings.

And now as to the practical result of this work, the inquiry is quite pertinent, as to what has been gained in the treatment of diseases of the Ear. To this, it may confidently be replied, in general terms, much has been done. To be more explicit, however, it can be said that the progress made in diagnosis has been eminently satisfactory. One of the most conspicuous instances in which this is shown, is in that class of diseases formerly designated as nervous deafness, the counterpart of which was once amaurosis, in Ophthalmology. Each being a convenient term, which was to embrace all that we did not understand regarding impaired hearing. That one prominent symptom of aural diseases, tinnitus aurium, which was supposed to be in so many of the cases a primary affection, is now known to be in many instances, owing to diminution or closure of the eustachian tubes, whereby the air in the middle ear becomes rarified, and the greater pressure from the more dense air upon the exterior of the membrana tympani, presses it in, upon the ossicles, and by modifying the undulations of sound produces the confusion of it known as tinnitus.

Another important fact ascertained is, that simple rupture of the membrani tympani does not mean total and permanent destruction of the drum, as was supposed by many, even members of the profession; for it is now known that such ruptures, in nearly all cases, unite readily, in the same manner as an ordinary incised wound. Hence the drum may often be punctured by the Surgeon with advantage, during severe inflammation of the middle ear, as in cases of scarlatina, where too often from want of familiarity with this fact, and from omission to watch properly the effect upon the ear, during the progress of that disease, hearing is greatly impaired in many instances, if not lost. Life itself, may even be saved by timely puncture of the drum and evacuation of fluid accumulation resulting from acute inflammation, in the cavity of the middle ear; otherwise the thin plate of bone dividing it from the brain may yield, from pressure, before suppuration shall have sufficiently destroyed the drum to allow free exit, and thus afford relief.

The supplying of artificial membrani tympani, in case of permanent loss of the natural one, is a valuable step in reducing the disadvantages resulting from the absence of that important portion of the accoustic apparatus, and often adds astonishingly to the ability of the sufferer to hear again, in addition to giving more or less complete protection from external influences, to a cavity, that, in its normal condition, does not communicate exteriorly with the atmosphere.

Another of the gratifying results of successful treatment of impaired hearing is in the improvement which is manifested in those cases of dulness of intellect, which are often an accompaniment of diminished hearing power, and apparently a consequence of it.

Ottorrhœa, which is mainly but a consequence of some preceding structural change, instead of a primary disease, has so often baffled surgeons in their attempts to control it, as to have justified the grave prognosis so many make. The possible results of the causes of otorrhœa are such that British Life Assurance Companies have deemed affections of the ear, resulting in otorrhœa, as adequate cause to preclude persons so affected from the benefits of assurance. One of the chief difficulties in the treatment of these cases, where the otorrhœa has been accompanied by only partial destruction of the membrana tympani, has, to a great extent, been remedied by Gruber of Vienna, who availed himself of the fact, that, in swallowing, the soft palate closes up against the (posterior part of the) pharynx,—obliterating, for the time, the passage down from the posterior nares to the throat—and the orifices of the Eustachian tubes open. At the instant that these two conditions are produced, he forces fluid, medicated as occasion may require, by means of a syringe, with blunt nozzle, fitting the nostril, through the nose and Eustachian tube into the middle ear. During the performance of this, of course, the other nostril is kept closed by pressure. By this means he is able to remove the accumulations from a larger surface of the middle ear than can be done by forcing water through the external meatus, and the orifice in the membrana tympani, for, by the latter method it is difficult to remove, from the inner aspect of the membrana tympani, the thickened and hardened secretions, and what is so attached is chiefly forced through the Eustachian into the throat, and is so offensive as to render it desirable to be avoided when possible. If violence be used in doing this, after Gruber's method, dizziness may be produced temporarily. To avoid anything of this kind, a syringe may be used with the Eustachian catheter, and nozzle properly adjusted to attach, thus to throw a direct stream through the Eustachian tube, with sufficient force to detach even moderately firmly adherent matter, and carry it out through the external meatus.

Investigation in this field of study was at first much impeded by the paucity of instruments and appliances suitable for its pursuit. Careful anatomical and physiological observation of the ear, enabled inventive genius to act intelligently, and soon the offspring of necessity became more numerous. Now we have apparatus for the exploration of the hidden mysteries of the ear, but little less efficient in revealing its interior than the opthalmascope is in the eye. Of these, Wild's Aural Speculum, designed to make straight the passage to the outer surface of the membrana tympani, and thus to show more plainly the appearance of that membrane, served its purpose well. Toynbee modified this somewhat, making the speculum conform more nearly, in its different parts, to the conformation of the auditory meatus. Other modifications of the same were made by others, but they have no marked advantage. All these were designed to be used with direct light, either natural or artificial, and to obviate certain inconveniences which attended their use, reflected light, after the manner of the light reflected from the mirror of the opthalmoscope, was introduced, which serves as a convenient mode of examination.

This speculum, used in connection with the mirror, might appropriately be called the simple otoscope.

A more efficient instrument, is what might, with less propriety, be called the compound otoscope, known by various names, as Clarke's, Anderson's, and others — each of whom claims to have added some improvement to it, making what is now a valuable instrument, constructed on much the same principle as the endoscope—light, either natural or artificial, being concentrated on a mirror, set at such angle as to throw the reflection through a Toynbee's speculum on the membrana tympani, whilst the observer looks through a perforation of the reflecting mirror, and gets a view which is magnified by a convex lens attached exteriorly.

Another instrument of recognized value, though of more limited scope of usefulness, is Siegler's pneumatic speculum, designed "to aid in determining the presence of bands of adhesion in the tympanic cavity, by indicating any spots at which the outward movement of the membrane might be impeded" (Hinton). It is constructed somewhat as a Toynbee's speculum, with its outer and larger orifice closed, and the inner and smaller one fitting tightly into the meatus externus. Through an opening in its side, the air may be exhausted, whilst an observer watches the effect upon the membrane, through the glass with which the outer opening is closed.

Politzer's aural manometer is "adapted especially for physiological demonstration of the vibrations of the air in the middle ear, as well as to show the permeability of the Eustachian tube, the influence of the acts of swallowing and respiration upon the membrana tympani, etc." "It consists of a horse-shoe shaped glass tube, $1\frac{1}{2}$ mm. in calibre, which is fastened in the auditory canal by means of a hard rubber nozzle smeared with grease. A drop of solution of carmine, contained in this aural manometer, indicates, by rising and falling, the variations in the pressure of the air in the auditory canal and cavity of the tympanum."

The aural douche, made as Thudichum's, or more appropriately, Weber's nasal douche, is a good substitute for the ordinary ear syringe, in inflamed and thinned conditions of the membrana tympani, and renders the dangers of perforation, by the stream, less.

Eustachian bougies, made of whalebone, are likewise serviceable in certain conditions of diminution of the calibre of the tube, and will be spoken of in connection with chronic catarrh of the middle ear. Wilde's polypus snare, and the polypus forceps, are not without their use, as well as Voltolini's galvano-caustic in removing foreign growths from the meatus.

Other instruments, devised for local treatment of the ear, embrace Moas' apparatus for generating vapor of muriate of ammonia, to be thrown through a Eustachian catheter into the middle ear, in cases of chronic thickening; Politzer's apparatus for the air-douche, in cases of temporary obstruction of the Eustachian tube; and the compression pump, designed also for the same purpose, but of less practical value, are now, comparatively little used.

Of the instruments for diagnosis mentioned, most were designed for investigation through the external meatus, but there are others of greater or less value, that are used to investigate through the Eustachian tube, either alone or aided by other and supplemental parts. Of these, perhaps the most important is the Eustachian catheter, valuable for its diagnostic utility, and its service in therapeutic use. Toynbee's "Explorer," and the diagnostic tube, separately, and in connection with the Eustachian catheter, are of great service.

The ordinary tuning fork of musicians is used with advantage in diagnosis in those cases where impaired hearing is dependent upon obstructed Eustachian tubes.

Of the many diseases an aural surgeon is called upon to treat in a variable climate, like that of much of the territory of the United States, where there are so great and so sudden changes of temperature, and often great humidity, probably the most frequent, and certainly one very important one, is chronic cataract of the middle ear, with the ordinary results, which are so manifold. The primary effects of acute catarrh are comparatively unimportant, except in so far as a thickened condition results, of the mucous and submucous tissues, and the consequent diminution of the calibre of the Eustachian tube. The acute symptoms attract, as a rule, but little special attention, but gradually, we find the size of the tube narrowing. The secretions of the mucous membrane, lining the whole interior cavity, become thickened, hardened, and changed, as we find in the mucous membrane of the nares. Thus, free exit to the fauces being precluded, the secretions are retained within the middle ear, in this altered and irritating condition, and thus effect and cause, act and react upon each other, until the whole membrane presents such thickened appearance, that in looking through the auditory meatus, the drum, instead of presenting its natural translucent appearance, appears opaque. The progress to this condition has generally been slow, almost unperceived, so gradual has it been. Perhaps but one ear is effected, or both may be, but the change from a condition of health has been so gradual, that the sufferer first becomes decidedly awakened to the altered condition, by his friends, who discover that he no longer hears so acutely, and, on closer observation, he finds that he is required to fix his attention more closely to discover the nicer distinction of sounds. He now finds that the occassional roaring noise that had been heard at times, occurs more frequently, and is so persistent that the simple act of swallowing will no longer relieve it, and air must be forced in with some energy, as by the Valsalvian method, before he obtains relief. We thus have the condition established, and the first effects apparent. As a consequence of the thickened condition of the drum, resulting from the additional thickness of the mucous membrane, and the adjacent tissues to which the inflammatory process had extended, it yields less readily to the undulations of sound. Perhaps the tube has become so much diminished in calibre, that with difficulty can air be forced in through it. As a consequence, the air in the cavity of the tympanum becomes rarified, and the drum becomes very conconcave inwards, and presses on the ossicles. Their natural free movement upon each other becomes impaired by this pressure, just as do the joints of the fingers, when the hand is encased in splints. The cartilage at the articulation with the fenestra, participates in the structural change which results from constant pressure, and absence of the natural movement. The bones, thus crowded together, become more firm, and form a more rapidly conducting medium for sound, and the supposed synchronous conduction of the sound through them, and through the atmosphere surrounding them, to the auditory nerve, is destroyed, and we may in this way account for the confusion of sounds which are heard under these circumstances, if the theory be accepted, of the two undulations of sound meeting at the apex of the cochlea, in normal condition, and thus neutralizing each other, as in the meeting of opposing waves in water, in the normal condition of the ear.

The results of the chronic inflammatory process, although mainly manifested in the middle ear, are not limited to that space. It is well known, how rapidly inflammation extends along mucous membranes, and hence the lining of the mastoid cells are affected, and the orifices of the Eustachian tubes may participate in the structural change, as the rhinoscope often reveals to be the case.

We have now seen the progress of the disease—some of the structural alteration of the tissues, and some of the natural consequences of those changes. In addition to the destruction of the harmonious action of the whole, as an acoustic apparatus, we have the effect of the mechanical obstruction of bloodvessels, which, in addition to the impaired hearing, adds to the mental obtuseness we often see attending these cases.

Now that we see the physical changes in the auditory apparatus of the patient and realize the disadvantage to him, the most important question to him is, what can we do to remedy them?

In our profession, we all realize the primary importance of obtaining a correct knowledge of the pathological change with which we have to deal. In this respect, I am persuaded that progress has of late been made, which augurs favorably for more satisfactory treatment of these cases. It were desirable that a greater degree of success in treating them were in our command; but because we have in some cases to acknowledge that only experimental trial can determine how much can be done for them, it should not discourage us to such an extent as to preclude effort, in even those cases, for the number is far from small, where marked benefit is given, if only the patient will persevere in treatment. If we do occasionally fail to accomplish our object with certain of our cases, even after patient effort, it is only the same result which attends many efforts in all the departments of medicine and surgery. Besides, the condition produced by this catarrhal inflammation does not remain stationary. If not treated, it continues to grow worse, and if we fail in our efforts at treatment to do more than stay progress of the disease, that is a result worth all the effort it costs, and is, in a degree, success.

How we must proceed to accomplish the result desired, circumstances must determine. The degree of structural change, the general health of the patient, the age, temperament, and other collateral considerations, will have a modifying influence. As in nasal catarrh, one important point is to get rid of the thick and altered mucous secretion which accumulates, from the orifices of the Eustachian tubes, through to the membrani tympani. For this purpose, I have found the steam atomizer, with a solution of chlorate of potassæ, or muriate of ammoniæ, effective — the salt in connection with warmth and moisture being serviceable. This however, presumes a degree of permeability of the Eustachian tubes. Where the calibre of them is so small as to require some force to propel air into them, by the introduction of the Eustachian catheter, and forcing of air in by means of a rubber inflator, the secretions may be loosened and even forced out by the return current. Politzer, of Vienna, seems to have been the first to have given practical recognition to the fact that in the act of swallowing, the soft palate closes up against the posterior wall of the pharynx, and at the same time the orifices of the Eustachian tubes open. Availing himself of this fact, he gives his patient a mouthful of water, and whilst in the act of swallowing it, an inflator which has been inserted into one nostril — the other being closed at the same time — pressure is made, and pure air is forced into the middle ear through the Eustachian tube, with great advantage in simple and recent cases. In other cases, requiring more decided impression by the forcing of iodized air in, by means of a Buttles' inflator, the requisite degree of stimulation may be obtained. To do this the nozzle of the inflator is inserted into one nostril, the other being closed, and the patient is made to swallow a mouthful of water at the same time that pressure is made, which will accomplish the desired end; if not, attaching the inflator to a Eustachian catheter and pressing the bulb will nearly always force a stream of the medicated air in. However, if this fail, we have still another resource in the Eustachian bougie, which may be introduced through the Eustachian catheter, having been annointed or not, as occasion may seem to require.

Frequently the application of stimulating or astringent substances to the orifices of the Eustachian tubes, proves of advantage, as auxiliary treatment.

In a large proportion of cases, this local treatment seems to be all that is required. There are others, though, where constitutional impression is required in addition. Not unfrequently we find accompanying this affection a debilitated and relaxed condition of the system, which, of course, indicates tonics along with alteratives. Of these, I prefer the bi-chloride of mercury in diminutive doses, in solution with the compound tincture of cinchonia, though at times I find a stronger tonic than bark desirable.

A careful examination of the properly managed Turkish bath satisfies me that it may be used with advantage in these cases. The free perspiration produced by the air, heated to a high degree, encourages the skin to healthy action, and tends in no small degree to equalization of the circulation. As to the practical value of counter-irritation over the mastoid cells, professional opinion is divided. The weight of testimony seems, however, to favor its use in certain cases; but it seems to require persistent use to be of service, and is apparently most beneficial in those cases where the inflammation has extended to the mastoid cells.

